# Diagnostic relevance of metastatic renal cell carcinoma in the head and neck: An evaluation of 22 cases in 671 patients

**DOI:** 10.1590/S1677-5538.IBJU.2015.0665

**Published:** 2017

**Authors:** Anja Lieder, Thomas Guenzel, Steffen Lebentrau, Constanze Schneider, Achim Franzen

**Affiliations:** 1 Department of Otorhinolaryngology, Ruppiner Kliniken and Brandenburg Medical School Theodor-Fontane, Neuruppin, Germany;; 2 Department of Otorhinolaryngology, Head and Neck Surgery, Borromaeus-Hospital Leer Germany;; 3 Department of Urology and Pediatric Urology, Ruppiner Kliniken and Brandenburg Medical School Theodor-Fontane, Neuruppin, Germany;; 4 Clinical Cancer Registry Brandenburg, Neuruppin, Germany

**Keywords:** Carcinoma, Renal Cell, Neoplasm Metastasis, Carcinoma, squamous cell of head and neck [Supplementary Concept]

## Abstract

**Purpose:**

Renal cell carcinoma (RCC) is a malignant tumor that metastasizes early, and patients often present with metastatic disease at the time of diagnosis. The aim of our evaluation was to assess the diagnostic and differential diagnostic relevance of metastatic renal cell carcinoma (RCC) with particular emphasis on head and neck manifestations in a large patient series.

**Patients and methods:**

We retrospectively evaluated 671 consecutive patients with RCC who were treated in our urology practice between 2000 and 2013.

**Results:**

Twenty-four months after diagnosis, 200/671 (30%) of RCC had metastasized. Distant metastases were found in 172 cases, with 22 metastases (3.3%) in the head and neck. Cervical and cranial metastases were located in the lymph nodes (n=13) and in the parotid and the thyroid gland, tongue, the forehead skin, skull, and paranasal sinuses (n=9). All head and neck metastases were treated by surgical excision, with 14 patients receiving adjuvant radiotherapy and 9 patients receiving chemotherapy or targeted therapy at some point during the course of the disease. Five patients (23%) survived. The mean time of survival from diagnosis of a head and neck metastasis was 38 months, the shortest period of observation being 12 months and the longest 83 months.

**Discussion and conclusion:**

Our findings show that while RCC metastases are rarely found in the neck, their proportion among distantly metastasized RCC amounts to 13%. Therefore, the neck should be included in staging investigations for RCC with distant metastases, and surgical management of neck disease considered in case of resectable metastatic disease. Similarly, in patients presenting with a neck mass with no corresponding tumor of the head and neck, a primary tumor below the clavicle should be considered and the appropriate staging investigations initiated.

## INTRODUCTION

Renal cell carcinoma (RCC) is a malignant tumour of the kidney that metastasizes early. Most commonly, metastases occur in lung, bone or liver and often in multiple sites (1). Head and neck metastases are rare but there is little evidence in the literature as to their pattern and management.

Numerous single case reports and small series of metastasis of RCC into the head and neck region are available in the literature. These case reports focus mainly on particular, unusual, and especially extranodal location of the metastases as well as unusual clinical courses (2-8). The aim of this study was to assess the differential diagnostic and also the therapeutic relevance of metastatic RCC in a large series of RCC and to evaluate if a systematic examination of the head and neck is appropriate in the context of staging RCC. We present an analysis on RCC metastasizing into the head and neck region based on a large group of 671 consecutive patients with an RCC treated in our unit.

## PATIENTS AND METHODS

Medical records of 671 consecutive patients who were diagnosed with an RCC in the Department of Urology of Ruppiner Kliniken, a large District General Hospital, between 2000 and 2013 were evaluated. All patients were followed-up until the time of their death; surviving patients were followed-up for at least 24 months from the date of diagnosis. All data were collected from case notes, anonymized and maintained in an Apache OpenOffice4 database and analyzed using a statistical software package (Apache OpenOffice4 Calc with R4Calc R extension). As this study was a retrospective case notes study, formal ethical approval was not required. Written consent was obtained from all patients prior to undertaking any procedures but for this retrospective case note audit, formal written consent was not required. All investigations and treatments were carried out according to accepted clinical practice and were compliant with the medical principles of the Declaration of Helsinki.

## RESULTS

Of 671 consecutive patients diagnosed with RCC, 200 (30%) had distant or regional lymph node metastases either at the time of diagnosis or within 24 months of diagnosis. The overall metastatic rate, including locoregional metastases, was 17% (111/671) at the time of diagnosis of RCC, with an additional 13% (89/671) diagnosed following treatment.

Distant metastases were found in 172 patients (26%), and regional lymph node metastases in 22 patients (3%). In 92 patients (14%), metastases were identified at the time of diagnosis of the primary tumour, and in the remaining 80 patients (12%) metastasis occurred over the course of the following 24 months despite curative intent treatment (Table-1).

**Table 1:** Patient and tumour characteristics.

No.	Age *	Histology	Grade	TNM at diagnosis	primary management	Time to Metastasis **	Location Metastasis	Other metastases	Management of metastatic disease	Survival after Metastasis (H&N) in months
1	70	clear cell	2	pT2bpNxM1	nephrectomy	0	Level IV Lymph node	Lung, bones	Excision	2
2	58	clear cell	2	pT3apN0M1	nephrectomy	5	Thyroid Gland	Retroperitoneum, pancreas	Excision (Thyroidectomy), Sunitimib	24 (patient alive)
3	74	clear cell	***	unknown	nephrectomy	78	Parotid Gland	none	Excision (Parotidectomy),	56 (patient alive)
4	52	clear cell/poly	3	pT3pN2 M1	nephrectomy	5	Level IV Lymph node	Lung, bones	Excision, radiotherapy, chemotherapy	14
5	78	clear cell	2	pT2NxM1	nephrectomy	65	Frontal Sinus	Brain, lung, bones, liver	Radiotherapy	2
6	69	undifferentiated	3	cT3cN1cM1	chemotherapy	0	Level IV Lymph node	Bone, liver, peritoneum	Excision, radiotherapy, chemotherapy	14
7	73	clear cell	2	pT1bNxMx	nephrectomy	94	Level IV Lymph node	Lung, bones	Excision, radiotherapy, chemotherapy, Sorafenib	7
8	69	clear cell	3	pT1cN0cM0	nephrectomy	14	Level IV Lymph node	Peritoneum	Excision, radiotherapy	4
9	70	undifferentiated	***	TxN1M1	chemotherapy	0	Level IV Lymph node	Lung, mediastinum	Chemotherapy, Sunitimib	28
10	69	undifferentiated	***	pT3cN0cM1	nephrectomy	24	Level IV Lymph node	Lung, bones, liver, adrenal glands	Excision, chemotherapy, Sunitimib	12
11	32	Nephroblastoma	3	pT3bpN0cM1	nephrectomy	0	Level IV Lymph node	Lung, liver	Chemotherapy, radiotherapy (other centre)	24
12	56	clear cell	3	pT3acN1cM1	nephrectomy	5	Tongue	Lung, bones, mediastinal nodes, soft tissue finger	Excision (glossectomy), radiotherapy	3
13	66	polymorph	3	pT1bNxMx	nephrectomy	75	Frontal Skull bone	Lung, bones, soft tissue back	Sunitimib, chemotherapy radiotherapy	13
14	81	undifferentiated	***	unknown	declined treatment	0	Level IV Lymph node	none	Declined treatment	0
15	69	clear cell	2	pT1acNxM0	nephrectomy	69	Level IV Lymph node	Retroperitoneal lymph nodes (paraaortal)	Excision, chemotherapy	19
16	68	clear cell	2	pT1bpN0cM0	nephrectomy	36	Facial Skin (forehead)	Lung, adrenal glands, jejunum	Excision	19 (patient alive)
17	48	clear cell	2	pT1bN0M0	nephrectomy adrenalectomy	87	Thyroid Gland	Lung, bones, mediastinum	Excision, laminectomy, chemotherapy, Sunitimib	27 (patient alive)
18	72	small cell	***	cT4cN1M1	resection metastasis	0	Frontal Skull bone and mandible	Lung, brain, mediastinal lymph nodes	Excision, radiotherapy	13
19	73	clear cell	2	pT1a cN0 M1	nephrectomy	40	Thyroid Gland	Lung, bones, mediastinum	Excision, radiotherapy	86 (patient alive)
20	78	clear cell	3	pT2bNxM0	nephrectomy	6	Level IV Lymph node	Lung, retroperitoneum	Excision, Sunitimib	7
21	63	undifferentiated	***	unknown	nephrectomy	0	Level IV Lymph node	Lung	Excision, none to lung	2
22	65	undifferentiated	***	unknown	unknown	unknown	unknown	unknown	unknown	Lost to follow up

*Patient age at the time of head and neck metastasis

**Time from first diagnosis of RCC to head and neck metastasis in months

***Tumour Grade undetermined

Metastases of RCC in the head and neck were found in 22 patients (3%). Sixteen patients were male and six were female. The mean age of these patients at the time of diagnosis was 66 years (32-81 years). In 10 patients (45%), head and neck metastases appeared simultaneously to the primary tumour, or the metastasis was the first manifestation of the RCC. In 12 patients (55%), metastases were detected at follow-up following curative intent treatment after 24 months on average. The longest period between treatment of the primary RCC and the detection of metastases in the Head and Neck was 87 months.

The histological type of renal cell carcinoma was clear cell renal carcinoma in 14 cases, poorly differentiated or undifferentiated carcinoma in six cases, nephroblastoma in one case and small cell renal carcinoma in one case. Tumour Grade was G2 in 8 cases, G3 in 9 cases, and undetermined in 5 cases. Initial TNM stages ranged from T1N0M0 to T3N2M1 at the time of diagnosis.

**Figure 1:**
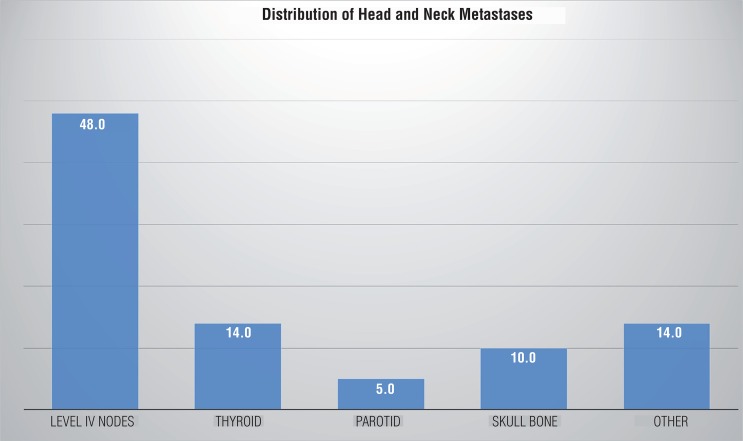
Distribution of head and neck metastases by location (in % of n=22 patients).

Metastases to cervical lymph nodes were found in 12 out of 22 cases. Organ metastases were found in the parotid (n=1) and thyroid gland (n=3) and skull bone (n=2). Other locations (n=3) included the tongue, facial skin and frontal sinus (Figure 1). Recurrence in context of a metachronous cervical metastasis was seen in one case. In 19 out of 22 patients, synchronous disseminated metastases were detected in other organs at some stage during the course of the illness. In 10 patients, this occurred simultaneously with the head and neck metastases. The most important metastatic target organs in these 19 cases were the lung (n=12) and the skeletal system (n=9). Other less frequent locations were the liver, the brain, and the peritoneum. In 3 out of 22 patients, metastasis occurred solely in the head and neck.

All 22 patients received curative intent treatment at the time of diagnosis, except for one patient, who declined treatment. Eighteen patients, all of whom had the primary tumour diagnosed first or synchronous with the head and neck metastasis, received a nephrectomy.

In the 18 cases where metastases in the head and neck were found after diagnosis of the primary tumour or at staging of the primary tumour, patients received curative intent treatment at the time of initial diagnosis and were then followed-up by either a hospital or community urology tumour surveillance programme.

Nephrectomy was performed in 17 patients, total nephrectomy in 13 patients and partial nephrectomy in 4 patients. When metastases of the head and neck occurred, they were treated by surgical resection and adjuvant radiotherapy. In the 10 patients where diagnosis of the RCC head and neck metastasis preceded (4 patients) or coincided (6 patients) with diagnosis of the primary tumour, patients received surgical treatment of the head and neck metastasis first followed by surgery to the primary tumour followed by adjuvant radiotherapy.

Radiotherapy was performed in 14 cases. Radiotherapy after primary tumour resection was performed in one patient, following resection of the head and neck metastasis in 4 cases, and following detection of other metastases in 9 cases. The dose of radiotherapy was 40 Gray except in four patients who requested palliative treatment; 25 Gray were administered in such cases.

Chemotherapy was performed in 9 patients, usually following the diagnosis of disseminated metastatic disease. Due to the long observation period, chemotherapy regimens changed over time and included both standard chemotherapy, chemoimmunotherapy and targeted therapy. In particular, targeted therapy with either Sunitinibe or Sorafenibe was given to 6 patients.

Sixteen patients (73%) with head and neck metastases died from RCC. The time of death was on average 25 months after an RCC was first diagnosed, and 13 months after diagnosis of a head and neck metastasis. The median survival time after a RCC was first diagnosed was 28 months, meaning that 11 patients (50%) were still alive at 28 months after their RCC was diagnosed. The median survival time after diagnosis of a head and neck metastasis was 13 months. Patients died from either disseminated disease or local recurrence with the exception of one case, who died from an acute event.

**Figure 2:**
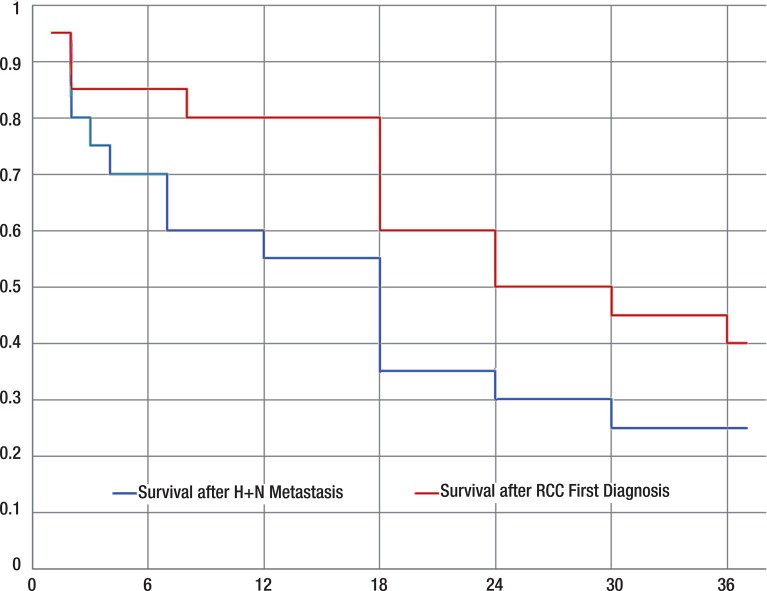
Survival after diagnosis of a head and neck metastasis, shown in months with standard deviation.

Five patients (23%) survived and one patient was lost to follow-up. The mean time of survival from diagnosis of a head and neck metastasis was 38 months, the shortest period of observation being 12 months and the longest 83 months (standard deviation 30 months) (Figure-2).

## DISCUSSION

RCC are slowly growing, capsule-forming tumours and most frequently metastasize into lung and the lymph nodes, followed by the skeletal system and the liver – in the majority of the cases, several organ systems are affected simultaneously (1). The metastatic rate of 17% (111/671) in our patient group at the time of diagnosis, and an additional 13% (89/671) in the further course of the disease, is lower than described in other studies (2). This could be explained by the fact that 66% of our cases had been diagnosed in an early stage (61% in stage I) and had been well differentiated (Grade 1 + Grade 2: 68%).

RCC are considered to be the third most frequent infraclavicular tumour metastasizing to the head and neck. Supraclavicular metastases were found in 3% (22/671) of all our patients with RCC. In the literature, there are reports of metastatic rates of up to 15% (3-6). Whether these results can be compared to those presented here, remains to be discussed with regards to size of study, stage of disease, duration of follow-up and whether all patients were staged specifically for metastases of the head and neck.

While the proportion of RCC metastasizing to the head and neck was low at 3%, we observed head and neck metastases in 11% (22/200) of all metastasized RCC and in 13% (22/172) of all distantly metastasized RCC.

According to our results, RCC metastasizing into the head and neck area primarily metastasize into the cervical lymph nodes. In the literature, there are several reports about unusual manifestations of RCC in different organs of the head and neck. Single case observations refer to the parotid gland, the skull, the skin, the oral cavity, and the paranasal sinuses (3, 5-10), which were also seen in our patient group. Two large multicentre studies also reported metastatic spread of RCC into the thyroid gland, a phenomenon that was also observed in two of our patients (10, 11).

Our observations also show the variable pattern of cervical metastasis of RCC. In some cases, a cervical metastasis may represent the first manifestation of an RCC, in other cases, cervical metastases may occur months or years after curative intent treatment of an RCC (5, 6, 12). In 3 out of 22 patients, a solitary cervical lymph node metastasis was the first manifestation of a previously unknown RCC. At the other end of the spectrum, a solitary metastasis appeared in the parotid gland 6 years after diagnosis of the primary tumour. In the other 19 patients, the metastatic spread of the RCC into the head and neck occurred at the same time as metastasis into other organ systems.

Lymph node metastases and metastases of the parotid gland generally occur as painless, relatively slowly growing tumours (7, 9, 13). Facial nerve palsies in conjunction with parotid metastasis of a RCC are rare (3). Metastases within the upper aerodigestive tract such as the oral cavity and the pharynx are often painful. They are usually diagnosed when patients present with sore throats or oral pain, and grow nearly always submucosally, show signs of increasing vascularization and are often distinguished from mucosa by their red discolouration. Such lesions will bleed profusely when biopsied or haemorrhage spontaneously, and life-threatening haemorrhage has been reported. Metastases in the supraglottic larynx may cause narrowing of the upper airway, stridor and dyspnoea. Manifestations of the nasal cavity or the paranasal sinuses lead to nasal obstruction, sinusitis-like complaints, or significant haemorrhage (14).

According to our observations, the head and neck were involved in 13% of distantly metastasized RCC. This must be considered in patients who are due to undergo extensive surgery of either the primary tumour or metastases in other locations. Appropriate staging procedures would include imaging of the neck by either computed tomography (CT) or magnetic resonance imaging (MRI) with contrast and, if upper aerodigestive tract symptoms are present, a laryngo-pharyngoscopy.

Surgery as therapeutic option of metastasized RCC has an great significance. Good oncologic clearance is achieved in particular if metastases occur more than two years after treatment of the primary tumour, and where there is good surgical access. This applies to large case series on treatment outcomes of lung and liver metastases of RCC (15-17), and international guidelines recommend surgical therapy of metastases despite improvements of chemotherapy outcomes (2). Larger series of surgical therapy of supraclavicular metastases have only been published for patients with thyroid gland metastases. The five-year survival rate of those patients amounted to 51% (10, 11). Only case reports only exist about the surgical therapy of RCC metastases in other supraclavicular locations. Curative therapeutic options exist in cases of single metastasis into the head and neck (7, 8), but surgical management of head and neck metastases can also be appropriate for symptom control in cases of airway obstruction, haemorrhage, or pain (13). We observed survival of 23% of patients with a head and neck metastasis following treatment, and would therefore have no hesitation in recommending curative intent management of head and neck metastases in all patients fit for surgery.

## CONCLUSIONS

Our results show that 3% (n=22) of all patients with an RCC (n=671) treated in our unit developed metastatic disease into the head and neck. This accounts for 9% of metastasized RCC. It remains open for discussion whether inclusion of the head and neck into the staging procedure should be recommended – it should, however, be considered in all cases of metastasized RCC. It is also of note that head and neck metastases of RCC may occur at any time during the course of the illness and any patient reports of head and neck-related symptoms such as neck swelling, sore throat, dysphagia or foreign body sensation should prompt an otolaryngologist examination and an ultrasound examination of the neck and thyroid at the very least, bearing in mind that while most metastases occur in supraclavicular lymph nodes, they may also present in an unusual location. Surgical management of such metastases should be considered in all patients fit for surgery for both curative intent and palliative treatment.
